# Tau Phosphorylation in Female Neurodegeneration: Role of Estrogens, Progesterone, and Prolactin

**DOI:** 10.3389/fendo.2018.00133

**Published:** 2018-03-28

**Authors:** Daniel Muñoz-Mayorga, Christian Guerra-Araiza, Luz Torner, Teresa Morales

**Affiliations:** ^1^Departamento de Neurobiología Celular y Molecular, Instituto de Neurobiología, Universidad Nacional Autónoma de México, Querétaro, Mexico; ^2^Unidad de Investigación Médica en Farmacología, Hospital de Especialidades, Centro Médico Nacional Siglo XXI, Instituto Mexicano del Seguro Social, Mexico City, Mexico; ^3^Centro de Investigación Biomédica de Michoacán, Instituto Mexicano del Seguro Social, Morelia, Mexico

**Keywords:** tau phosphorylation, estrogen, progesterone, prolactin, hippocampus, neuroprotection, neurodegenerative disease, reproduction

## Abstract

Sex differences are important to consider when studying different psychiatric, neurodevelopmental, and neurodegenerative disorders, including Alzheimer’s disease (AD). These disorders can be affected by dimorphic changes in the central nervous system and be influenced by sex-specific hormones and neuroactive steroids. In fact, AD is more prevalent in women than in men. One of the main characteristics of AD is the formation of neurofibrillary tangles, composed of the phosphoprotein Tau, and neuronal loss in specific brain regions. The scope of this work is to review the existing evidence on how a set of hormones (estrogen, progesterone, and prolactin) affect tau phosphorylation in the brain of females under both physiological and pathological conditions.

## Introduction

Tau protein, named after its ability to induce tubule formation (1), is a phosphoprotein that is almost exclusively found in neurons, and it has six molecular isoforms derived from the alternative splicing of a single gene (2). Tau is mostly found in the axon (3, 4), but its presence has also been reported in dendrites, oligodendrocytes, and astrocytes (5–7).

The relevance of tau in a number of neurodegenerative diseases, especially Alzheimer’s disease (AD), has been widely documented (8). When in hyperphosphorylated state (p-Tau), tau aggregates to form neurofibrillary tangles (NFTs), a hallmark of AD (9). Currently, the triggers and threshold for tau to change into an aggregated pathogenic promotor are not well understood (10). Since only a small percentage of AD cases have a genetic background, research has led to the identification of risk factors, among the most studied are: age (the greatest risk factor), proneness to experience stress (11, 12), anxiety and depression (13), head injury, lack of physical exercise, obesity (14), low education level (15), and sex (16).

Sex differences are present across several psychiatric, neurodevelopmental, and neurodegenerative disorders, including AD (17, 18). Moreover, females undergo hormonal changes throughout life, which affect p-Tau (19). Among these changes, reproductive conditions such as puberty, the use of hormonal contraceptive methods, length of reproductive life, and pregnancy at late age have shown to correlate with better cognitive performance in postmenopausal stage (20). More importantly, breastfeeding is related to a reduced risk to develop AD, especially when performed for a prolonged period (21). Likewise, maternal experience results in better performance in hippocampus-dependent learning tasks (22) and acute stress during lactation induces a decrease in hippocampal p-Tau compared to unstressed controls in rats (23). The following sections will focus on the role of a specific set of hormones (i.e., estrogen, progesterone, and prolactin) and its consequences for p-Tau.

## Regulation of Tau Phosphorylation

Tau phosphorylation is not only relevant to AD, but it is also a process that occurs in physiological conditions. The most emblematic function of tau is the ability to bind and stabilize microtubules by copolymerization with tubulin. This function is tightly regulated by the phosphorylation state of tau: when in a more dephosphorylated state, tau is more efficient at promoting microtubule formation (24). As a consequence of this hallmark function, tau also participates in axogenesis, axonal transport, neurite extension processes (25, 26), and coordinated phosphorylation and dephosphorylation within the microtubule has been proposed as a step for neurite outgrowth (27).

Tau phosphorylation is a dynamic process that relies on the interaction of different kinases (enzymes adding phosphate groups to serine, S; threonine, T; and tyrosine, Y) and phosphatases (enzymes removing phosphate groups from S, T, and Y) (28). In spite of the large number of kinases that phosphorylate tau protein, just a few have been implicated as prominent players in abnormal tau processing *in vivo*, such as glycogen synthase kinase 3β (GSK3β) and cdk-5 (29). Regarding phosphatases, tau can be dephosphorylated by PP1, PP2A, PP2B, and PP5 (28–30). Phosphatase activity in normal brains is due to PP2A in 71% and PP2B in 11% of the cases, making PP2A a major brain and tau phosphatase (30). In AD, the activity of PP2A is reduced by half and that causes tau hyperphosphorylation and memory deficits, while increasing GSK3β activity (28).

**Figure 1:**
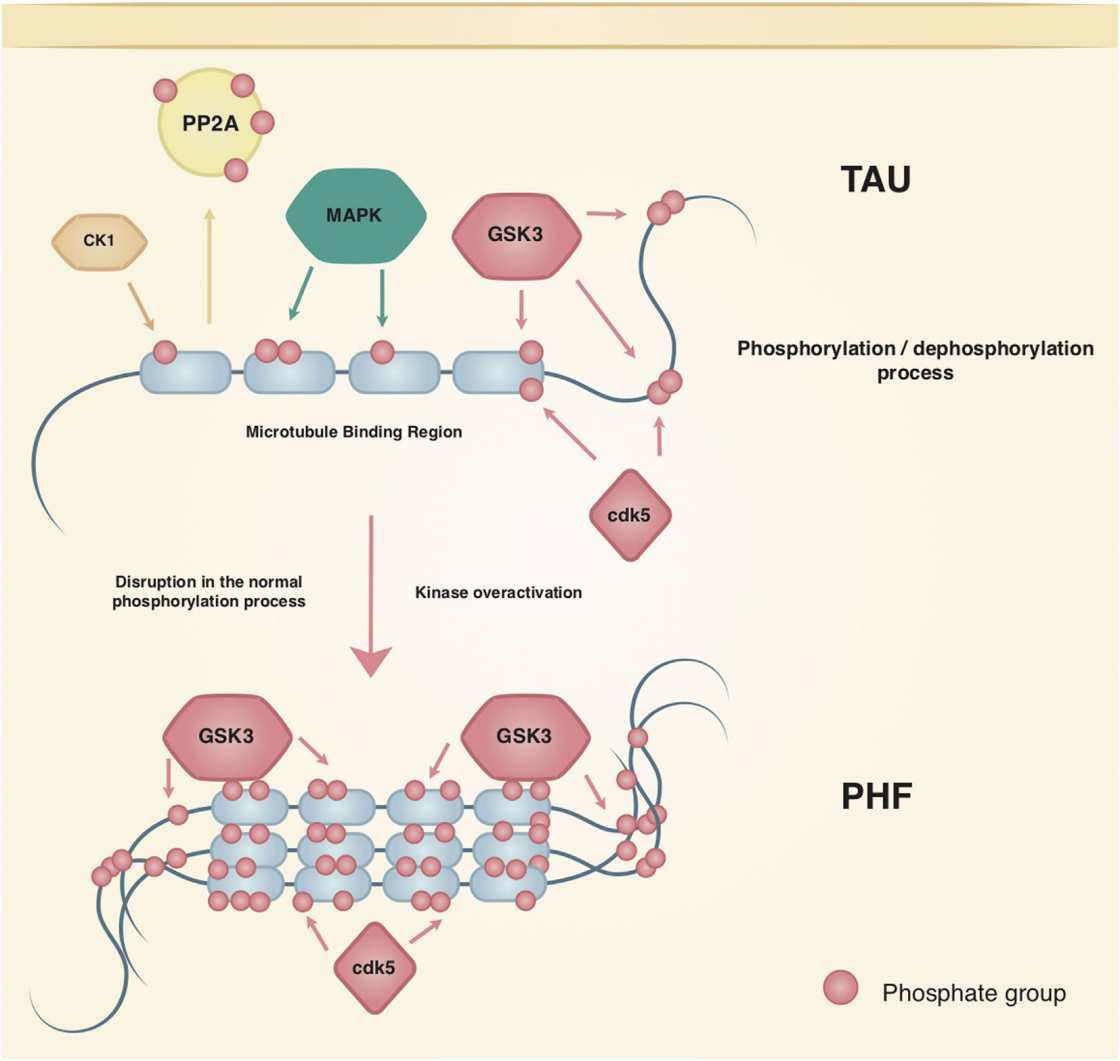
Regulation of tau phosphorylation. Tau is a native phosphoprotein that relies on an equilibrium of phosphorylation and dephosphorylation to perform physiological and neuroplasticity processes. Tau most studied kinases and phosphatase are shown; kinases such as cdk5 and GSK3 have been related to the hallmark lesions in Alzheimer’s disease.

The most studied tau kinases in the field of AD are GSK3, cdk5, mitogen-activated protein kinase (MAPK) (p38, ERK1/2, JNK), CK1, and MARK. GSK3 is able to phosphorylate 42 sites of tau, 29 of which are found in brains with AD (31). GSK3 refers to two homologous proteins that are paralogs: GSK3α and GSK3β. The catalytic sites of both are identical except for a glycine-rich N-terminal region in the GSK3α, which is absent in the GSK3β paralog. The mechanisms regulating their expression are not well understood and some differential actions in synaptic plasticity and disease are known. More research is needed to clarify the role of each paralog in different physiological and pathological pathways (Figure 1) [see Ref. (32) for review].

P-tau has been implicated in neuronal plasticity. For example, its most phosphorylated isoform is expressed in fetal stages (2), reversible and transient hyperphosphorylation occurs during hibernation and deep anesthesia in squirrels (33), and pregnancy and lactation alter tau expression and p-Tau in rats (34). Regarding AD, cognitive and memory impairment have been correlated with synapse loss in the prefrontal cortex (35) and in other brain areas (36) of AD patients, along with presence of tau NFTs (37). Immunization of 3xTg-AD mice against the N-terminal domain of tau decreased p-Tau and reduced the cognitive deficits in reference memory, as observed in the Morris water maze (38).

Studies using GSK3β show that hippocampal degeneration is mediated by p-Tau, which presents colocalization with the aforementioned NFTs. Besides, the increased GSK3β activity by AB peptides also causes tau hyperphosphorylation in the hippocampus of rodents (31), while inhibition of GSK3β can protect neurons from AB toxicity. Moreover, once cdk5 phosphorylates tau, it primes the protein to be more efficiently phosphorylated by GSK3β (31) showing that interactions among enzymes that regulate p-tau should be taken into account (Figure 1).

## Hormone Actions and Their Relation to Neurodegenerative Diseases and Tau Phosphorylation

Epidemiological studies show a significantly higher prevalence of AD in women than in men, which is usually explained by the longer lifespan of women (39). A considerable amount of research shows that the higher frequency in women may be due to the interplay between age and sex, in which, factors such as genetics, metabolic changes, and hormones play a prominent role (40). Ovarian hormones, such as estrogens and progesterone, could interact with other risk factors (obesity, neuroinflammation, mitochondrial dysfunction, etc.) to develop AD (15, 41).

The notion of female sex as a risk factor for AD (16, 40) is also supported by studies using transgenic mouse models of AD where sex differences were documented, suggesting that females are more vulnerable to the neuropathology (16). In addition, the hippocampal response to stress shows that females are more vulnerable to tau (42) and AB pathology (43) than males.

In line with this, menopause, characterized by the loss of estrogens and progesterone due to aging, is strongly associated with a higher vulnerability to develop AD (16). The absence of ovarian hormones increases the age-induced p-Tau in the hippocampus of rats (44). Regarding estrogens, plasma levels of 17β-estradiol are lower in women with AD compared with age-matched controls and some estrogen-based approaches to reduce AD risk have been designed, although with contradictory results (19). Progesterone, also depleted in menopause, has known effects in AD neuropathology (45). Progesterone and its metabolites can exert neuroprotective actions by themselves (19, 46), although it can potentiate or block the protective effects of estrogens. In rats treated with estrogens, progesterone blocked estrogen-induced spatial memory improvement and neuroprotection from excitotoxic injury, among others. Such antagonistic mechanisms are not yet fully understood (19).

On the other hand, there is a strong correlation between reproductive history and cognition in postmenopausal women. Particularly, women who had their last pregnancy later in life showed better verbal and global cognitive performance (18). Furthermore, mothers outperform nulliparous rats in learning and memory tasks (47), and these positive changes may endure into senescence (19). During their lifetime, most females undergo radical physiological changes induced by the maternal experience (47). Hormones such as prolactin, oxytocin, and endorphins produce these changes, which fundamentally alter the functions of the HPA axis (48).

For such changes in the female brain to occur during the reproductive experience, a great deal of plasticity is required (47). This plasticity translates into changes in the cytoskeleton and microtubule-associated proteins, for example, Tau content in the hippocampus decreases throughout pregnancy, but the ratio of phosphorylated tau increases in pregnancy and lactation (24, 46). Estrogen, progesterone, the interplay between them, and other hormones (49, 50) are known to be involved in those changes.

### Estrogens

**Figure 2:**
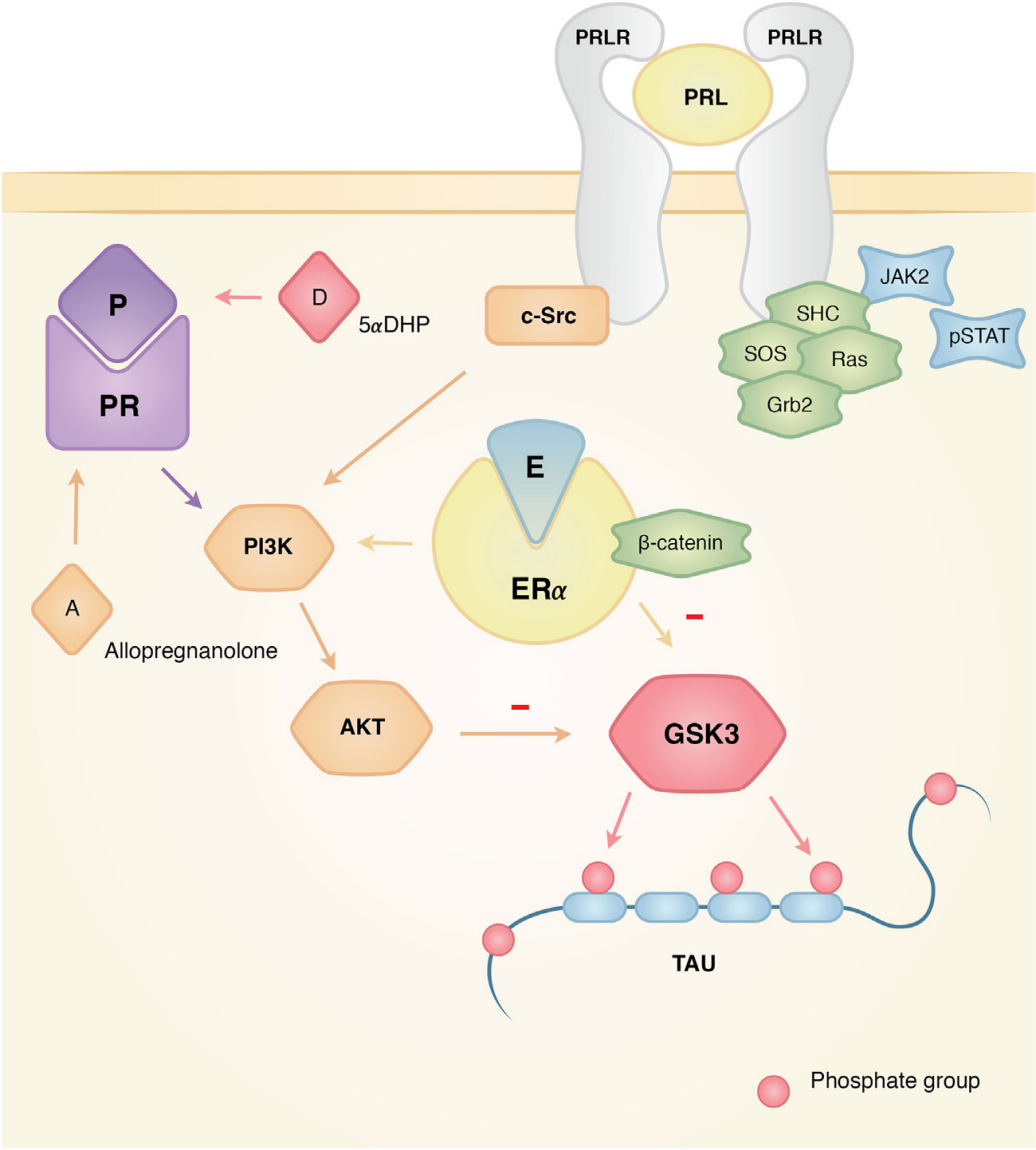
Actions of estrogen, progesterone, and prolactin on tau phosphorylation. Interacting mechanisms of hormone action that affect the dynamics of tau phosphorylation. The three hormones have documented activity in the activation of Akt that makes GSK3 inactive, which results in the inhibition of tau phosphorylation for this particular kinase. Since GSK3 has been linked in the pathological development of Alzheimer’s disease (AD), the interplay between these hormones, their pathways, and GSK3 might explain why the absence of hormones could account for the higher risk of developing AD. On the other hand, the presence of hormones like prolactin might prove neuroprotective for the neurodegeneration of the female brain.

Estrogens have long been known to exert neuroprotective effects in different models of central nervous system diseases such as AD, Parkinson’s disease, and multiple sclerosis (51). Estrogens may exert their neuroprotective properties through estrogen receptor α (ER-α), which is known to interact with insulin-like growth factor 1 receptor (IGF-1R), by incorporating itself into a macromolecular complex with the components of IGF-1R signaling (49). These include phosphoinositol 3 kinase (PI3K), protein kinase B (Akt), GSK3β, and β-catenin. The activation of PI3K and Akt results in inhibition of GSK3β (by phosphorylation of the site Ser9), therefore, reducing p-Tau (52, 53) (Figure 2).

In the hippocampus of ovariectomized rats sacrificed 1 h after treatment with estradiol, GSK3 phosphorylation increases significantly in Ser^9/21^, a site that inactivates GSK3. In addition, there is less phosphorylation in the PHF-1 epitope, which recognizes phosphorylation in Ser^396/404^, a site observed in AD brains, both *in vivo* and *in vitro*. It was also observed that β-catenin, a key protein regulated by GSK-3 and located in the Wnt/wingless pathway, co-immunoprecipitated with ERα. These results support the idea that a multi-complex between ER-α, β-catenin, and GSK3 is formed to modulate the activity of GSK3, and in turn, tau phosphorylation, through the Wnt and Akt pathways (52, 54).

In N2a cell cultures (neuroblastoma cell line) treated with Wortmannin and GF-109203X to induce GSK3β activation and p-Tau, the treatment with 17β-estradiol resulted in attenuated p-Tau at sites Ser^396/404^, Thr^231^, Thr^205^, and Ser^199/202^ (55). Furthermore, when a transient deactivation of Akt (the upstream regulator of GSK3β) was induced, 17β-estradiol increased the levels of GSK3β phosphorylated in Ser^9^, which suggests that estrogens can also target GSK3β directly and affect tau phosphorylation without the upstream effector Akt, in addition to the pathway described above (55).

*In vitro* studies in human neuroblastoma and primary rat cortical neuron showed that treatment with estradiol increased tau dephosphorylation as assessed with Tau-1 antibody, which recognizes a proline-directed non-phosphorylated site of tau (56). Moreover, when tau hyperphosphorylation was induced by okadaic acid in differentiated human neuroblastoma cells, treatment with estradiol for 24 h decreased p-Tau measured by 12E8 tau antibody (which recognizes phosphorylation in Ser^262^ within the microtubule-binding region and is not a proline-directed site) (56). Estrogen treatment also affected p-Tau in both undifferentiated and differentiated neuroblastoma cells (56). According to this study, the ER receptor seems to be involved in these effects, likely *via* the Akt pathway [see above, Ref. (52, 53)].

On the other hand, when using *in vivo* models of AD, the neuroprotective actions of estrogens are mainly related to AB production and clearance [see Ref. (45) for review]. Nevertheless, in a study using 3xTg-AD transgenic female mice that develop the complete AD neuropathology, the ovariectomized group that was treated with estrogens and progesterone showed a robust decrease in AT8 immunoreactivity, which detects tau phosphorylated at sites Ser/Thr^202/205^, in hippocampal neurons. This result shows that the combination of the hormones can regulate AD neuropathology and their absence could be deleterious (57).

Finally, when ovariectomized female rats were chronically treated with estrogen, progesterone, and tibolone (TIB) (a widely prescribed synthetic steroid with estrogenic, progestogenic, and androgenic actions), less p-Tau in the epitope PHF-1 and more dephosphorylation in the antibody Tau-1 was detected, in both the hippocampus and the cerebellum (58). In this particular study, no changes were detected in GSK3β Ser^9^ in the hippocampus, in contrast to the findings in the cerebellum, suggesting the involvement of different pathways or a region-specific action for this enzyme.

### Progesterone

Like estrogens, progesterone is a hormone that is mainly synthesized in the ovary and exerts neuroprotective effects in several models of disease such as AD, stroke, and traumatic brain injury (59, 60). Progesterone can elicit its neuroprotective effects through progesterone receptors (PR) A and B. Interestingly, PR-A can also modulate the activity of PR-B, ER (which might explain its antagonistic or synergistic effects when administered with estradiol) and the glucocorticoid receptor. It has also been shown that progesterone can activate signal transduction pathways such as cAMP/PKA, MAPK (ERK1/2), and PI-3K/Akt (60) (Figure 2).

In relation to tau, progesterone, and its metabolites, DHP (5-alpha-dihydroprogesterone) and THP (3-alpha, 5-alpha-tetrahydroprogesterone, also known as allopregnanolone) have been shown to significantly reduce tau protein expression in the rat cerebellum (46). Moreover, progesterone induces tau phosphorylation in the epitope Tau-1 and PHF-1 (sites Ser^396/404^) in the same structure, but this effect is not present when DHP or THP are administered. Since the epitope Ser262 (not phosphorylated by GSK3) was unaffected by progesterone treatment, while the epitope PHF-1 (phosphorylated by GSK3) responded to the same treatment, this implies that GSK3 is a possible candidate for the described progesterone effects (46).

Also, in the study by Carroll (57) mentioned earlier, p-Tau decreased significantly when ovariectomized female 3xTg mice were treated with progesterone and a progesterone/estrogens cocktail, as observed by immunoreactivity to the AT8 antibody in CA1 and subiculum subfields of the hippocampus. Based on these results, the authors suggest that the observed effect could be due to the regulation of GSK3β by progesterone (46).

On the other hand, treatment with physiological levels of progesterone 24 h prior to sacrifice increased the expression of the catalytic and regulatory subunits of PI3K and the phosphorylation of Akt in the hypothalamus, hippocampus, and cerebellum of ovariectomized rats. This effect was partially mimicked by progesterone-reduced metabolites DHP and allopregnanolone in the hypothalamus and hippocampus, but not in the cerebellum (61). This study provides a likely pathway for the decrease in p-Tau by Akt phosphorylation, upstream to the inactivation of GSK3β in both cerebellum (46) and hippocampus (57). Also, progesterone increases the levels of the phosphatase PP2A phosphorylated in Y^307^, which inactivates this enzyme, in hippocampus and cerebellum (62).

It is worth noting that when estradiol was given prior to progesterone, an increase in Y^307^ phosphorylation was seen only in the cerebellum, which suggests that the regulation of PP2A by progesterone is region specific (62) and that a complex interplay between estrogens and progesterone regulates kinases and phosphatases (61, 62). Furthermore, exposure to either continuous or cyclic treatment of progesterone induced significantly less AT8 immunoreactive neurons in the 3xTg-AD model compared to their respective controls (63).

In ovariectomized female rats exposed to a 60-day treatment of estrogens, progesterone, or TIB, all compounds resulted in a significant decrease of p-Tau in the PHF-1 epitope and in higher levels of dephosphorylated tau in the hippocampus, as measured with Tau-1 antibody. However, progesterone had no effect in the cerebellum, and no differences in Ser^9^ phosphorylation were seen in GSK3β (58). Previous studies have shown reduced phosphorylation in the cerebellum coupled with changes in GSK3β activity after an acute treatment with progesterone (46). However, the chronic administration of progesterone that results in less phosphorylation could be mediated by other kinase mechanisms, such as ERK or cdk5, or by the involvement of phosphatases such as PP2A (58, 62).

A recent study using low and high doses of TIB showed decreased p-Tau in the PHF-1 epitope in the hippocampus of male aged rats through the regulation of PI3K/Akt and cdk5/p35/p25 pathways (64). Whether these pathways also mediate the action of TIB in the hippocampus of females is unknown. It is important to mention that both allopregnanolone and TIB have been proposed as neuroprotective agents, and that allopregnanolone is currently considered as a candidate therapeutic agent in patients with AD and postpartum depression, another risk factor of AD (13, 65–67).

### Prolactin

Prolactin is a pituitary hormone that is primarily involved in milk synthesis and maternal behavior (68). Beyond these well-known functions, it has been shown that prolactin provides neuroprotection to the hippocampus in the kainic model of epilepsy (69, 70) and attenuates the neuroendocrine responses to stress (71, 72). There is evidence that breastfeeding lowers the risk to develop AD in humans (21), and tau expression undergoes changes during pregnancy along with an increase in P-Tau in several brain areas of rats throughout pregnancy until day 2 of lactation (27). These data point to the maternal experience as a regulator of tau phosphorylation, being prolactin a likely candidate for promoting these changes due to its elevated concentration during lactation and its actions inhibiting the HPA axis (72). We found that exposure to one episode of restraint stress significantly reduced p-Tau in the hippocampus of lactating dams compared to virgin or postweaned rats. This decrease was coupled with reduced detection of GSK3α (23).

Given that lactation is considered a hyperprolactinemic state, it is possible that the elevated levels of prolactin affect signaling pathways related to kinases that regulate p-Tau (Figure 2). Previous studies using male mice subjected to the same stress protocol showed increased p-Tau insolubility in the hippocampus (73), which reinforces the view that prolactin could regulate aspects of tau phosphorylation in the hippocampus.

Prolactin exerts its effects through prolactin receptors (PRL-R) *via* long, intermediate, and short isoforms encoded by a single gene. After homodimerization of the PRL-R, long–long homodimers can activate second messenger pathways, particularly the JAK–signal transducer and activator of transcription signaling cascade. Short–short homodimers activate the MAPK pathway and, finally, long and short heterodimers are known to block PRL-R signaling and modulate its effects (74).

Although a direct link between prolactin and p-Tau has not been investigated, evidence from prolactin pathways involving GSK3β suggests that the hormone influences p-Tau. In W53 lymphoid cells, prolactin increases the activity of the Akt pathway, thereby phosphorylating GSK3β in Ser9 causing its deactivation (75). On the other hand, a study using breast cancer cell lines identified GSK3β as a kinase of the prolactin receptor at Ser349; this phosphorylation site labels the prolactin receptor for degradation (76).

## Future Perspectives

The fact that diverse results in the AD field have surfaced between males and females at different ages, reproductive stages, and in response to different stimuli points toward a new direction in sex-related AD research (23, 45, 77) (Table 1). Thus, detailed attention must be given to the study of aging, different reproductive stages and hormones involved, and the interplay with other risk factors in females (21, 77).

**Table 1:** Phosphorylation sites in tau influenced by estrogen, progesterone, and prolactin.

Hormone	Phosphorylation site	Reference
Estrogens	Ser396/404	(54–57)
	Ser262	
	Ser202/Thr205	
	Thr231, Thr205, Ser199/202	
Progesterone	Ser396/404	(46, 57, 63)
	Ser202/Thr205	
Prolactin*	Ser396/404, Ser202/Thr205*	Steinmetz et al.* (23)

*It must be noted that the evidence about prolactin influencing these phosphorylation sites is not direct and needs more research.

A considerable amount of studies shows that estrogens and progesterone play an important role in regulating p-Tau in different conditions. However, the evidence about prolactin influencing p-Tau is still missing despite the importance of this hormone in reproductive life. Moreover, it is important to have thorough knowledge of how puberty, the estrous cycle, pregnancy, lactation, and maternal experience affect p-Tau, since all of these life events are characterized by hormonal fluctuations that cause fundamental changes in the female’s brain.

Also, it is extremely important to acknowledge that inflammation plays a critical role in the development of AD, through interactions with risk factors, such as obesity and depression, and with the hormones described above (13, 15, 41, 78). Further research needs to address inflammation in relation to other risk factors and markers of the disease (12, 15, 23) to elucidate why females appear to be protected from injury and neurodegeneration during lactation and more vulnerable during menopause.

## Author Contributions

DM-M, CG-A, LT, and TM wrote and edited the review article. DM-M and TM created the figures.

## Conflict of Interest Statement

The authors declare that the research was conducted in the absence of any commercial or financial relationships that could be construed as a potential conflict of interest.
